# Follow-up of curatively treated cancer in primary care: a qualitative study of the views of Dutch GPs

**DOI:** 10.3399/BJGP.2021.0519

**Published:** 2022-07-12

**Authors:** Geertje B Liemburg, Joke C Korevaar, Wouter TG van Zomeren, Annette J Berendsen, Daan Brandenbarg

**Affiliations:** Department of General Practice & Elderly Care Medicine, University Medical Center Groningen, University of Groningen, Groningen.; NIVEL Netherlands Institute for Health Services Research, Utrecht, the Netherlands.; Department of General Practice & Elderly Care Medicine, University Medical Center Groningen, University of Groningen, Groningen.; Department of General Practice & Elderly Care Medicine, University Medical Center Groningen, University of Groningen, Groningen.; Department of General Practice & Elderly Care Medicine, University Medical Center Groningen, University of Groningen, Groningen.

**Keywords:** breast cancer, colorectal cancer, follow-up care, general practice, qualitative research, shared care

## Abstract

**Background:**

Follow-up for cancer typically occurs in secondary care, and improved survival has increased demands on these services. Other care models may alleviate this burden, such as moving (parts of) follow-up care for curatively treated patients from secondary to primary care (care substitution).

**Aim:**

To explore the opinions of GPs regarding the potential benefits, barriers, and requirements of care substitution for breast and colorectal cancer.

**Design and setting:**

A qualitative study of the opinions of purposively sampled GPs in Dutch primary care.

**Method:**

Focus group sessions and individual semi-structured interviews were recorded and transcribed verbatim. Data were analysed by two independent researchers using thematic analysis.

**Results:**

Two focus groups (*n* = 14) were conducted followed by nine individual interviews. Three main themes were identified: perceived benefits, perceived barriers, and perceived requirements. Perceived benefits included better accessibility and continuity of care, and care closer to patients’ homes. Uncertainty about cancer-related competences and practical objections were perceived as barriers. Requirements included close specialist collaboration, support from patients for this change, and stepwise implementation to avoid loss of existing care quality.

**Conclusion:**

Most GPs reported that they were not in favour of complete care substitution, but that primary care could have greater formal involvement in oncological follow-up if there is close collaboration with secondary care (that is, shared care), support from patients, sufficient resource allocation, stepwise implementation with clear guidelines, and monitoring of quality. Clear and broadly supported protocols need to be developed and tested before implementing follow-up in primary care.

## INTRODUCTION

The incidence and prevalence of cancer is rising in developed countries because of population ageing, earlier cancer detection, more favourable staging at detection, and improved treatment.^1^^,^^2^ Breast cancer is the most diagnosed malignancy worldwide among females, whereas colorectal cancer ranks third for both males and females.^2^ Although both breast and colorectal cancer are leading causes of death, the number of cancer survivors has increased as the associated mortality rates have decreased over the past decade.^1^^,^^3^

After curative treatment, patients typically receive follow-up over a 5-year period to detect recurrence early, monitor the side effects and long-term effects of treatment, and provide psychosocial support.^4^^,^^5^ Given that this care is mostly hospital based, the increasing number of people living with cancer has led to a greater demand on healthcare resources in these settings. Many countries, including the Netherlands, are now debating whether other follow-up models could alleviate this burden while maintaining or improving the quality and patient-centredness of care. The Dutch College of General Practitioners has stated that follow-up care for the most prevalent types of cancer (breast cancer, colorectal cancer, lung cancer, prostate cancer, and melanoma) could be moved (partially) from secondary to primary care (care substitution), provided evidence-based protocols are available for use by GPs.^6^ Research indicates that care substitution does not diminish safety, health-related quality of life, psychological wellbeing, or patient satisfaction in primary care.^7^^–^9 Dutch GPs have no formal role in cancer follow-up care, but studies indicate that patients consult their GP more often during this period.^10^^–^14 Qualitative studies have also shown that patients consult GPs for more information about their cancer, lifestyle advice, and psychosocial issues during treatment and follow-up.^15^^,^^16^ A systematic review of qualitative studies reported that GPs supported a greater role in follow-up care for people living with cancer; however, conditions such as better communication, easy referral options, and clear guidelines are necessary.^17^ Several studies have tried to identify models of care, but there is still disagreement about their formats and implementation strategies.^18^

Although there appears to be room to integrate cancer follow-up in the care already provided by GPs, more formal involvement would be informed by the views of all involved parties. Despite qualitative evidence of the feasibility and acceptability of follow-up care in primary care according to GPs, there are limited research data available about how GPs think such care should be offered.^19^^–^21

**Table:** How this fits in

Primary care involvement in oncological follow-up could alleviate increases in demand for healthcare resources in secondary care. Currently, GPs are not formally involved in this follow-up care in the Netherlands. Previous qualitative studies have identified the perceived general requirements for follow-up care substitution among GPs. The current study considered how Dutch GPs thought these requirements could be applied in practice to help lift the burden on secondary care while maintaining quality of care and patient-centredness.

The current study explores the opinions of GPs in the northern and middle regions of the Netherlands regarding the potential benefits, barriers, and requirements of care substitution, with a view to inform possible future oncological follow-up strategies. Care substitution does not necessarily mean that all aspects of follow-up are substituted; it may also be applicable only for select parts (certain procedures) of follow-up care. The focus was on follow-up care substitution after curative treatment for patients with breast or colorectal cancer. Follow-up care in this study refers to both cancer surveillance for recurrence of cancer and aftercare.

## METHOD

### Study design

A qualitative study was undertaken of primary healthcare providers (mostly GPs). A grounded theory approach based on an interpretivist–constructivist paradigm was used.^22^ A hybrid approach of inductive and deductive coding was used.^23^ As relevant literature already exist a deductive approach was used to inform the topic list. The themes in this topic list (Box 1) were also used as starting themes for the thematic analysis. Thereafter, an inductive approach was used to identify new themes and codes that emerged during analysis of the interviews.

**Box 1.** Topic list used to guide semi-structured interviews

Concerning the follow-up care provided to patients curatively treated for breast cancer and colorectal cancer, the following were asked: What is the current role of GPs regarding follow-up care? What do GPs think about the feasibility of follow-up care in primary care? What opinions do they have about performing (parts of) follow-up care in primary care? ○ What are the perceived benefits and barriers? ○ Which follow-up tests do GPs think they can perform? ○ What are the requirements for effective care substitution? For which patient groups could GPs coordinate follow-up care? What would GPs need to be able to perform (parts of) follow-up care? If oncological follow-up is to be (partially) introduced in primary care:^a^ What would GPs consider the ideal organisation of cancer follow-up care in primary care? ○ How should implementation be structured? ○ Which stakeholders are involved? ○ How would GPs organise it within their own practice? ○ Who is going to do what? ○ How do GPs think we can overcome resistance? ○ What is necessary for successful cooperation with secondary care?

a *Topics that arose during the interviews (that is, an iterative process).*

### Participants and recruitment

An invitation letter for study participation was sent to a large group of GPs (*n* = 150) from 51 practices, via the Academic General Practitioners Development Network (AHON). The AHON committee assessed the recruitment plan for the study and gave permission to use their database. GPs affiliated with the AHON gave permission to be approached for participation in scientific research. First, GPs in the AHON database were purposively selected by age, sex, practice type (single handed/group), area (urban or rural), and location in the Netherlands. A random sample of these GPs was invited by mail, followed by a telephone reminder after 2 weeks. After the first series of focus groups/interviews, Dutch GPs at the 24th WONCA Europe Conference for Family Medicine were purposively sampled to collect a wider range of opinions across the Netherlands. The participants and researchers had no relationship before the study.

### Data collection

The study and data reporting were performed according to the consolidated criteria for reporting qualitative research checklist (COREQ).^24^^,^^25^ First, focus groups were performed in which participants were encouraged to share and discuss their opinions to create a dynamic discussion. Subsequently, semi-structured individual interviews were conducted to gain more in-depth information. A topic list was developed based on a literature review and the clinical expertise of the research group (Box 1).^16^^,^^26^^,^^27^ Face-to-face focus groups were performed by an experienced interviewer and focus group leader, assisted by two observers. Individual interviews were conducted by two of the authors (the first author and the senior author) at a location most convenient to the participant. The first author received training in qualitative methods and was trained in performing interviews by the senior author. Field notes were made during the interviews. The focus groups and interviews were audio-recorded, transcribed verbatim (by two other authors), and pseudonymised. Audio-recordings were checked for inaccuracies in transcription and to enhance semantic understanding by the first author. Short questionnaires were sent to all participants to collect demographic information, and all data were entered into a data management program (REDCap 8.10.18, Vanderbilt University, Tennessee, USA).

### Data analysis

Thematic analysis was performed by two researchers^28^ who independently marked relevant transcript segments (inductive coding) and identified new codes and themes. Discrepancies were discussed until consensus was reached, and, if this was not possible, an independent researcher made the final decision (open, axial, and selective coding). When new topics arose, these were used in the topic list for new interviews (that is, an iterative process). This was repeated until no new themes emerged from four consecutive transcripts, indicating data saturation. A member check (that is, responder validation) of the in-depth interviews of four randomly selected GPs was undertaken to assess internal validity by sending a summary of their interview to check for accuracy and whether it resonated with their experiences. A member check was only performed among some participants because of efficiency and the authors believed that in this manner it was possible to avoid putting an unnecessary strain on all participants.

Data were coded in ATLAS.ti version 8.4. IBM SPSS version 26 was used to analyse the characteristics of participants.

### Ethical considerations

The Medical Ethics Review Committee of the University Medical Center Groningen concluded that this study was not subject to the requirements of the Dutch Medical Research Involving Human Subjects Act. All participants gave written informed consent after the study and procedures had been fully explained before the interviews, and their data were pseudonymised by allocating them unique numerical identifiers and stored according to privacy regulations.

## RESULTS

### Participants and interviews

In total, 22 medical GPs and a single GP-based nurse (special interest in oncology) participated in this study (hereinafter, all are referred to as GPs). Seventeen GPs responded after a personal invitation (response rate, 11%) and five were recruited by purposive sampling at the 24th WONCA Europe Conference; the GP-based nurse was recruited by a GP as she had received oncological training and was currently implementing elements of oncological aftercare in their practice. The participant characteristics are presented in Table 1. Their mean age was 52 years (range, 35–66 years), 65% (15/23) were male, and they had 3–36 years’ experience.

**Table 1.** Characteristics of the interviewed GPs (*n* = 23)

**ID**	**Sex**	**Age, years**	**Experience, years**	**Part-time factor, %^a^**	**Place of occupation**	**Type of GP practice**	**GP trainer**	**Affinity with oncology**	**Type of interview**
GP01	Male	38	8	100	Rural	Duo practice	No	Yes	Individual
GP02	Male	41	10	100	Suburban	Group practice	No	Yes	Individual
GP03	Male	41	9	100	Suburban	Group practice	Yes	Yes	Individual
GP04	Male	46	10	100	Suburban	Group practice	Yes	Yes	Individual
GP05	Male	66	26	100	Rural	Single-handed practice	No	No	Individual
GP06	Female	58	22	60	Suburban	Single-handed practice	Yes	Yes	Individual
GP07	Male	50	20	80	Urban	Single-handed practice	Yes	Yes	Focus group 1
GP08	Female	60	25	60	Rural	Single-handed practice	Yes	Yes	Focus group 1
GP09	Male	52	20	80	Urban	Single-handed practice	Yes	Yes	Focus group 1
GP10	Male	65	36	100	Urban	Group practice	Yes	Yes	Focus group 1
GP11	Female	46	14	70	Suburban	Group practice	Yes	No	Focus group 1
GP12	Male	62	34	100	Urban	Single-handed practice	Yes	No	Focus group 1
GP13	Male	53	21	80	Suburban	Duo practice	Yes	Yes	Focus group 1
GP14	Male	64	22	60	Rural	Group practice	Yes	No	Focus group 1
GP15	Female	61	30	80	Rural	Single-handed practice	Yes	Yes	Focus group 1
GP16	Male	66	28	100	Rural	Single-handed practice	Yes	Yes	Focus group 1
GP17	Female	35	3	60	Urban	Locum	No	No	Individual
GP18	Female	54	25	70	Suburban	Group practice	Yes	Yes	Focus group 2^b^
GP19	Male	56	25	80	Suburban	Group practice	No	Yes	Focus group 2^b^
GP20	Female	45	16	78	Suburban	Employed GP	No	Yes	Focus group 2^b^
GP21^c^	Female	47	7	60	Suburban	Group practice	NA	Yes	Focus group 2^b^
GP22	Male	39	8	80	Urban	Group practice	No	Yes	Individual
GP23	Male	50	16	40	Urban	Group practice	No	Yes	Individual

a *The percentage of hours worked compared with full-time employment.*

b *Participants in focus group 2 were from the same GP practice.*

c *General practice nurse with an interest in oncology. NA = not applicable.*

Two focus group sessions were conducted before moving on to nine individual interviews, all of which took place between April and December 2019. The first focus group included 10 GPs all from different practices and took place at a centrally located hospital (duration 70 mins) and the second included four GPs from the same practice and took place at their practice (duration 40 mins). The interviews were face to face (except for two individuals) and took place at GP practices (duration 23–35 mins).

After the focus groups and five individual interviews, no new themes emerged in that specific transcript and data saturation was assumed; this assumption was confirmed as no new information was obtained by analysing the next four consecutive transcripts. In the member check, all four randomly selected GPs agreed with the authors interpretations.

### Feasibility of oncological follow-up care in primary care

Most GPs indicated that they currently have no formal role during follow-up, except a few who provided individual follow-up care at the request of a medical specialist. Two GPs performed this follow-up: the first provided care for an older female patient for whom the hospital visits were too burdensome and the second GP provided care for a patient with a psychotic disorder who only trusted his GP. In their experience, easy consultation with the oncologist was a necessity to be able to provide such care.

Opinions about how this follow-up care could be offered in primary care varied enormously, from full GP provision to hospital-led follow-up, with different models of follow-up in between. Two contrasting but equally split viewpoints were identified on feasibility. One was that oncological follow-up care could be incorporated into current practice:

*‘We are a general practitioner throughout life … you lose sight of your patient during active treatment in hospital …* [then], *I often have to figure out how to keep in touch and with what frequency. It would be good to take over care when people start going back to their daily life after treatment. Primary care is the place for that care, not secondary care. So, I think it fits our profession.’*

(GP20, female, 45 years old)

The other viewpoint was that oncological follow-up care was not a primary care task. GPs expressed doubts about the effectiveness in general or were just not enthusiastic about performing protocolised care:

*‘Protocol-based care is not my favourite kind of care because it is literally and figuratively* [about] *ticking boxes, whereas I became a general practitioner to solve the puzzles and problems of patients.’*

(GP02, male, 41 years old)

### Themes

Thematic analysis identified three main themes: ‘perceived benefits’, ‘perceived barriers’, and ‘perceived requirements’.

### Perceived benefits

**Figure 1.**
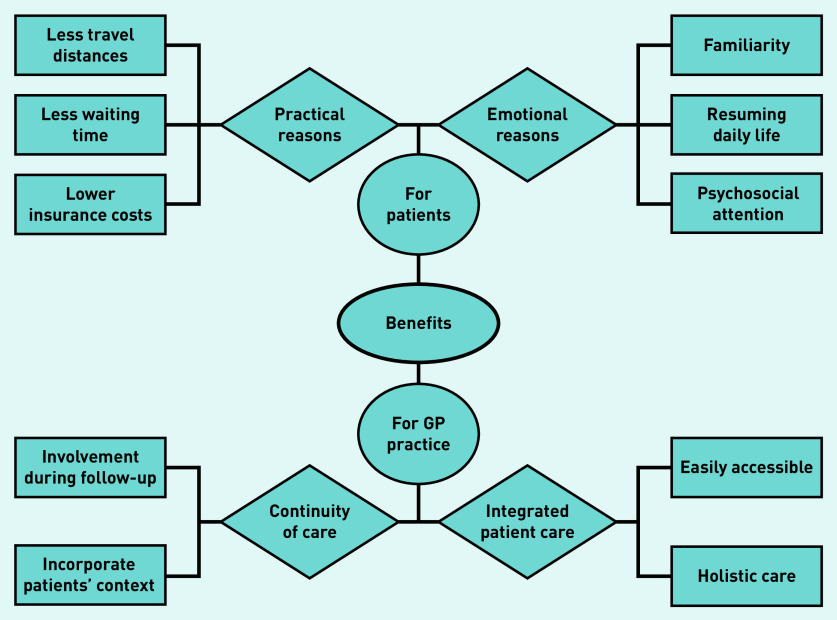
*Perceived benefits according to GPs.*

All participating GPs mentioned that involvement of GPs during follow-up had benefits. Overall, GPs made a distinction about benefits for patients and benefits for the GP practice specifically, which showed overlap. Sub-themes for patients were practical and emotional reasons, and the benefits for GP practices were continuity and integrated patient care (Figure 1). A frequently cited benefit was closer patient contact regarding their cancer.

In the focus groups, the main thought was that care substitution could bring care closer to patients’ homes, reduce referral waiting times, and save costs by not obliging them to use insurance deductibles (practical reasons). Furthermore, most GPs suspected that patients would be less anxious in a more familiar setting. Individually interviewed GPs added that familiarity and personal knowledge helps patients to open up about psychosocial issues. In addition, the majority saw a role for themselves in helping survivors in resuming daily life. Some considered older people with comorbidities, those living far from the hospital, and individuals who avoided care to be most suitable for follow-up in primary care, but most reported no preferences:

*‘The hospital can be perceived as a symbol for cancer, death, misery, and bald heads … Sick people go to a hospital, whereas a health centre is for people who are essentially healthy. So, this can be perceived as a kind of transition from a sickness to a healthier environment.’*

(GP20, female, 45 years old)

Another benefit identified in the focus groups was personal involvement because of continuity of care and comprehensive care. Most GPs, in both focus groups and individual interviews, indicated that their personal knowledge of the patient would help them to integrate patients’ needs in cancer care. They expressed that more involvement would help them better support patients, as they currently often feel insufficiently informed by secondary care. Some GPs also mentioned that they feel they were better suited to provide accessible care and looked beyond cancer-related issues compared with hospitals:

*‘Another advantage, in addition to being close to people’s homes, is the personal contact and that I know much more about the patient than only the oncological problem. This can probably result in disease-specific advantages, as you can have a more personal conversation.’*

(GP17, female, 35 years old)

### Perceived barriers

**Figure 2.**
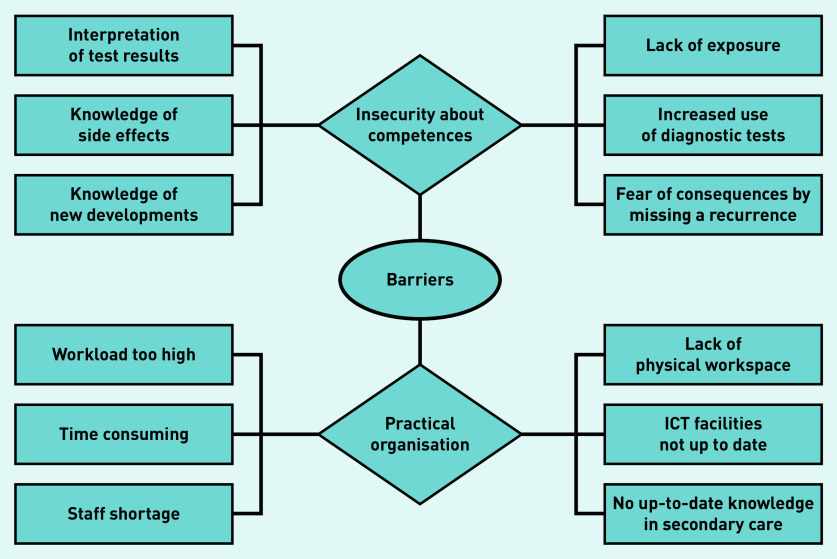
*Perceived barriers according to GPs. ICT = information and communication technology.*

During the focus groups, knowledge and capacity issues were the first topics of discussion. Most GPs were uncertain about their cancer-related competences and the practical organisation of a new care model (Figure 2). Many felt that their cancer knowledge of follow-up routines, interpreting tests, side effects, and long-term treatment effects were not up to date and that they may lack the required experience. Individual interviews added that they considered their caseload of patients with cancer to be too low to maintain up-to-date knowledge and keep up with new developments so as to be able to answer all questions. It was thought that this might lead to additional diagnostic tests because of a fear of missing disease recurrence:

*‘I think specialists are more perceptive for oncological problems than us, because we provide more universal care and they are very specific. Sure, we can interpret obvious signals, like CEA* [carcinoembryonic antigen] *that rises from being low: then we can notice something is wrong. But to interpret other things, like PET* [positron emission tomography] *scans, that is way too specialised and difficult.’*

(GP05, male, 66 years old)

Almost all mentioned that capacity issues are a bottleneck. Care substitution from secondary to primary care has occurred in other disciplines in recent years and according to GPs they have reached their limits. Most GPs mentioned that follow-up care substitution in oncology would increase their workload, perceiving current workload as already too high and leaving limited room for additional work.

Some GPs also mentioned logistic issues, such as lack of staff, physical workspace, or information exchange limitations such as suitable technological facilities for monitoring patients via scheduled appointments. Another perceived barrier was that care substitution might reduce a specialist’s up-to-date knowledge of a patient, limiting the ease of consultation between primary and secondary care. Some GPs feared that care substitution was merely proposed as a solution to reduce healthcare costs, fearing that they would receive insufficient remuneration:

*‘In fact, I don’t see how this is possible, because we are already fully occupied in terms of space for consultation rooms. Besides, if more care is substituted to general practice, consultation between primary and secondary care will become more difficult … they will see the patient less, or not at all … whereas now we both know a given patient, and that often facilitates the contact. This could be a negative change.’*

(GP22, male, 39 years old)

### Perceived requirements

**Figure 3.**
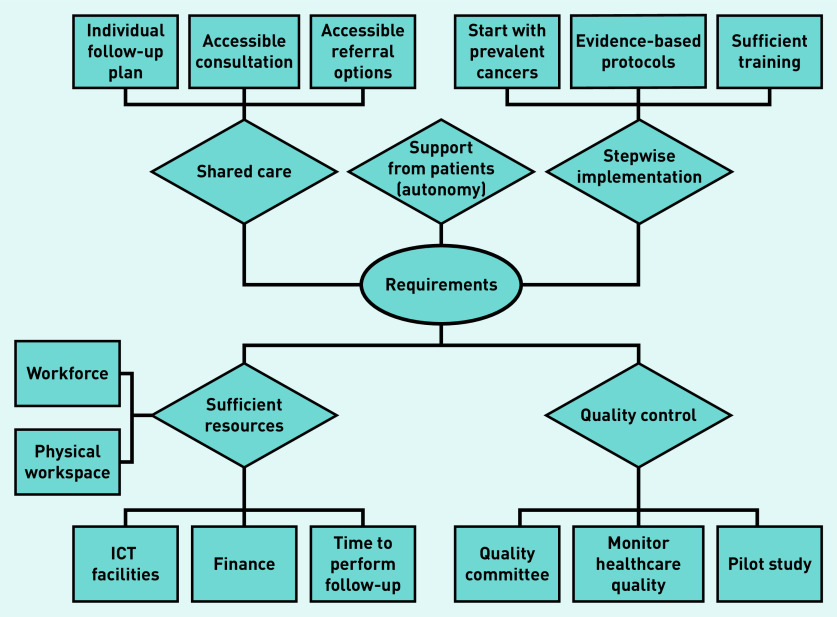
*Perceived requirements according to GPs. ICT = information and communication technology.*

During both focus groups and individual interviews, GPs mentioned a wide range of requirements. Some requirements were solutions to perceived barriers. Five sub-themes were identified, including shared care, support from patients, stepwise implementation, quality control, and sufficient resources (Figure 3).

During focus groups most GPs were willing to consider formal involvement in cancer follow-up if there was close cooperation with a specialist (that is, a shared-care model), support, and clear agreements. During individual interviews, GPs proposed an individual follow-up plan with information about side effects, a follow-up scheme, and allocation of tasks, for each patient as a requirement. They also mentioned that accessible re-referral options, and easy routes of consultation when a recurrence is suspected, should be in place.

Most GPs believed that support from patients for this change is crucial, and that they should retain autonomy to choose their preferred follow-up model. However, GPs typically thought that most patients will eventually get used to follow-up in primary care if it becomes the new norm:

*‘I think that if you make good agreements about how this follow-up care can be substituted, that it is not only possible in hospitals but also in GP practices. This should be discussed very carefully between the specialist and the patient … what is and is not possible … and ultimately, the patient should decide what to do and what not to do.’*

(GP03, male, 41 years old)

Most GPs deemed careful preparation and monitoring to be absolute necessities for stepwise implementation. They proposed ensuring adequate training and the development of clear evidence-based and up-to-date protocols, the latter ideally designed in collaboration with specialists. However, most wanted to know first which follow-up tests are really effective and evidence based. Almost all suggested starting with one high-prevalent cancer type. Some GPs suggested training a GP-based nurse or a GP with specialisation in oncology to perform the follow-up care. Most GPs preferred care substitution for procedures that are already part of their daily job, such as medical history, physical examination, ordering and interpreting blood tests, and ordering radiological examinations such as mammography:

*‘I’m willing to provide follow-up care, but first I want to know which research is useful. Subsequently, you can develop a roadmap including the problems to be expected, time points, and specific follow-up tests* […] *Thereafter, tasks can be divided between GPs and specialists.’*

(GP05, male, 66 years old)

It was also acknowledged that care provision should be monitored to ensure ongoing quality (quality control). Most recommended starting with a pilot study among all relevant stakeholders, such as primary and secondary care healthcare providers, patient associations, and health insurance companies:

*‘If you see cancer as a chronic disease and organise follow-up in line with how it is already organised for other chronic diseases, you will require facilitation with some aspects plus appropriate financing and education … That can be carried out in integrated care programmes, which is not a problem.’*

(GP06, female, 58 years old)

All GPs required sufficient resources to finance the necessary increase in staff, physical workspace, information and communication technology (ICT) facilities, and time to perform follow-up care. Almost all GPs commented on their outdated ICT facilities and expressed a wish for upgraded systems in which patients would be invited automatically for appointments according to their follow-up scheme. The majority thought that time might be an issue, as they believe these consultations would take more than the usual 10 minutes. Finally, some GPs proposed developing integrated care programmes for oncology, which in the Netherlands exist for chronic diseases:

*‘I think that secondary care should also invest time and space for communication with primary care. I don’t know how substitution will eventually look, but it will probably become a collaboration with alternating check-ups in the GP practice, and perhaps occasionally, still in secondary care.’*

(GP21, female, 47 years old)

## DISCUSSION

### Summary

Some GPs agreed that follow-up care for breast and/or colorectal cancer could be incorporated into current practice if certain requirements were fulfilled, but others were less excited about the proposed model for follow-up care. GPs perceived that care substitution could have benefits, such as improved continuity and integration of patient care, more psychosocial attention, and easier access because of familiarity and care close to patients’ homes. However, most felt uncertain about their cancer-related knowledge and skills, were reluctant because of capacity issues and outdated ICT facilities including digital information exchange with secondary care. However, future care substitution was considered possible if certain requirements could be met, including close collaboration with specialists (that is, a shared-care model), support from patients for this change, stepwise implementation, sufficient resources, and close monitoring of care provision to ensure quality of care.

### Strengths and limitations

The authors consider the combination of focus groups and individual interviews to be a strength, as it facilitated discussions between GPs as well as in-depth one-to-one conversations. A limitation is the risk of selection bias because of a low response rate. This could mean that only GPs interested in this topic responded to the invitation to participate, with the consequence that the results might not be generalisable to all GPs. However, this risk is considered to be minimal as both proponents and opponents of more involvement in cancer follow-up care were included and viewpoints on feasibility were about equally split. Another limitation is that participants could have expressed socially desirable responses; however, this should be minimal as the results show GPs not only cited benefits but also made critical remarks about follow-up care being provided by primary care.

### Comparison with existing literature

In line with the results in the current study, earlier studies have mentioned a number of requirements for moving cancer follow-up care from secondary to primary care. These included the need for clear coordination, clearly defined responsibilities, clear protocols, fast referral options, and appropriate financial compensation.^19^^–^21^,^^29^ Whereas these studies described general requirements, the current study provides more detail, as shown in Figure 3, such as close cooperation, attention to individual follow-up, stepwise implementation, close monitoring, and adequate resource allocation.

Some GPs in the current study were concerned that their limited cancer-related competences were barriers to perform oncological follow-up care, which is in line with other studies that show that GPs appear willing but they reported barriers and unmet needs related to providing such care.^30^ However, a recent Cochrane review and systematic reviews indicated that recurrence rate, survival, diagnostic delay, and patient satisfaction were not significantly different between follow-up in primary and secondary care.^7^^,^^31^^,^^32^ This shows that follow-up for patients with certain cancer types such as breast and colorectal cancer in primary care is safe regarding clinical outcomes and does not reduce quality of life for patients.

Some barriers still lacked solutions, with GPs in the current study unable to suggest how to manage the communication challenges, lack of ICT facilities, and increased workload. This is in line with other studies showing that communication challenges still exist between primary and secondary care, and that effective and timely communication is important if the situation is to be improved.^20^^,^^21^^,^^29^^,^^33^ Regarding the increased workload, patients with breast and colorectal cancer already consult their GPs significantly more often during follow-up for side effects and psychosocial issues,^10^^,^^11^^,^^34^^,^^35^ with evidence that one-third of these patients are already involved in a chronic disease management programme.^36^ This indicates that scheduled follow-up visits might be incorporated with this healthcare use, presenting an opportunity to improve psychosocial care.

### Implications for research and practice

The opinions of both hospital specialists and patients should also be explored regarding follow-up care substitution. This research could then be followed by a quantitative study in which preferences about the role of GPs in oncological follow-up care are studied, with the aim of developing evidence-based and supported protocols for collaborative cancer follow-up. Performing follow-up in primary care appears safe and feasible, but, to date, it still has not been widely implemented.^32^ Based on the suggestions of GPs in this study, and findings from other studies,^19^^–^21^,^^29^^,^^30^ it is possible to speculate how this might occur.

First, evidence-based protocols with clear agreements should be developed between primary and secondary care, preferably including arrangements for fast re-referral to, and consultation with, hospital specialists. However, before possible implementation, it would be interesting to investigate the evidence in the current guidelines, as studies have shown that intensive follow-up routines confer no survival benefit compared with less intensive follow-up.^37^^,^^38^ Second, GPs must have access to sufficient training and guidance. Third, implementation should be done in a stepwise manner, taking care to respect individual patient preferences. It was notable that GPs seemed most comfortable with the elements of follow-up care they already practise daily (for example, history taking, physical examination, and blood tests). In practice, the GP could perform all follow-up for breast cancer, but, for colorectal cancer, a shared-care model seems more feasible, in which blood tests and physical examinations are performed in general practice and imaging is performed in hospital.^39^ Finally, care quality should be monitored, and, when necessary, the model should be adjusted.

In conclusion, in this qualitative study, it was found that most Dutch GPs think that primary care could be involved more formally in oncological follow-up care, provided the new model can ensure close specialist collaboration, support from patients, sufficient resources, stepwise implementation, clear guidelines, and quality monitoring. Of note, clear and broadly supported protocols will need to be developed and evaluated before such a model can be implemented.

## References

[1] Arnold M, Sierra MS, Laversanne M (2017). Global patterns and trends in colorectal cancer incidence and mortality. Gut.

[2] Bray F, Ferlay J, Soerjomataram I (2018). Global cancer statistics 2018: GLOBOCAN estimates of incidence and mortality worldwide for 36 cancers in 185 countries. CA Cancer J Clin.

[3] Althuis MD, Dozier JM, Anderson WF (2005). Global trends in breast cancer incidence and mortality 1973–1997. Int J Epidemiol.

[4] Cardoso F, Kyriakides S, Ohno S (2019). Early breast cancer: ESMO Clinical Practice Guidelines for diagnosis, treatment and follow-up. Ann Oncol.

[5] Labianca R, Nordlinger B, Beretta GD (2013). Early colon cancer: ESMO Clinical Practice Guidelines for diagnosis, treatment and follow-up. Ann Oncol.

[6] Dutch College of General Practitioners (Nationaal Huisartsen Genootschap) (2014). NHG-Standpunt Oncologische zorg in de huisartsenpraktijk.

[7] Lewis RA, Neal RD, Williams NH (2009). Follow-up of cancer in primary care versus secondary care: systematic review. Br J Gen Pract.

[8] Grunfeld E, Gray A, Mant D (1999). Follow-up of breast cancer in primary care vs specialist care: results of an economic evaluation. Br J Cancer.

[9] Wattchow DA, Weller DP, Esterman A (2006). General practice vs surgical-based follow-up for patients with colon cancer: randomised controlled trial. Br J Cancer.

[10] Brandenbarg D, Roorda C, Groenhof F (2017). Primary healthcare use during follow-up after curative treatment for colorectal cancer. Eur J Cancer Care (Engl).

[11] Roorda C, Berendsen AJ, Groenhof F (2013). Increased primary healthcare utilisation among women with a history of breast cancer. Support Care Cancer.

[12] Brandenbarg D, Roorda C, Groenhof F (2014). Increased primary health care use in the first year after colorectal cancer diagnosis. Scand J Prim Health Care.

[13] Roorda C, de Bock GH, van der Veen WJ (2012). Role of the general practitioner during the active breast cancer treatment phase: an analysis of health care use. Support Care Cancer.

[14] Duineveld LA, van Asselt KM, Bemelman WA (2016). Symptomatic and asymptomatic colon cancer recurrence: a multicenter cohort study. Ann Fam Med.

[15] Roorda C, de Bock GH, Scholing C (2015). Patients’ preferences for post-treatment breast cancer follow-up in primary care vs. secondary care: a qualitative study. Health Expect.

[16] Brandenbarg D, Roorda C, Stadlander M (2017). Patients’ views on general practitioners’ role during treatment and follow-up of colorectal cancer: a qualitative study. Fam Pract.

[17] Meiklejohn JA, Mimery A, Martin JH (2016). The role of the GP in follow-up cancer care: a systematic literature review. J Cancer Surviv.

[18] Howell D, Hack TF, Oliver TK (2012). Models of care for post-treatment follow-up of adult cancer survivors: a systematic review and quality appraisal of the evidence. J Cancer Surviv.

[19] Duineveld LA, Wieldraaijer T, Wind J (2016). Primary care-led survivorship care for patients with colon cancer and the use of eHealth: a qualitative study on perspectives of general practitioners. BMJ Open.

[20] van Leeuwen A, Wind J, van Weert H (2018). Experiences of general practitioners participating in oncology meetings with specialists to support GP-led survivorship care: an interview study from the Netherlands. Eur J Gen Pract.

[21] Schütze H, Chin M, Weller D, Harris MF (2018). Patient, general practitioner and oncologist views regarding long-term cancer shared care. Fam Pract.

[22] Fereday J (2006). Demonstrating rigor using thematic analysis: a hybrid approach of inductive and deductive coding and theme development. Int J Qual Meth.

[23] Braun V, Clarke V (2006). Using thematic analysis in psychology. Qual Res Psychology.

[24] O’Brien BC, Harris IB, Beckman TJ (2014). Standards for reporting qualitative research: a synthesis of recommendations. Acad Med.

[25] Tong A, Sainsbury P, Craig J (2007). Consolidated criteria for reporting qualitative research (COREQ): a 32-item checklist for interviews and focus groups. Int J Qual Health Care.

[26] Heins M, Schellevis F, Schotman M (2018). Feasibility and acceptability of follow-up for prostate cancer in primary care: a pilot study. BJGP Open.

[27] Berendsen AJ, Roorda C, Jansen L, de Bock GH (2016). Patients’ beliefs about the aims of breast cancer follow-up: a qualitative study. Maturitas.

[28] Pope C, Ziebland S, Mays N (2000). Qualitative research in health care: analysing qualitative data. BMJ.

[29] Easley J, Miedema B, Carroll JC (2016). Coordination of cancer care between family physicians and cancer specialists: importance of communication. Can Fam Physician.

[30] Lawrence RA, McLoone JK, Wakefield CE, Cohn RJ (2016). Primary care physicians’ perspectives of their role in cancer care: a systematic review. J Gen Intern Med.

[31] Høeg BL, Bidstrup PE, Karlsen RV (2019). Follow-up strategies following completion of primary cancer treatment in adult cancer survivors. Cochrane Database Syst Rev.

[32] Vos JAM, Wieldraaijer T, van Weert HCPM, van Asselt KM (2021). Survivorship care for cancer patients in primary versus secondary care: a systematic review. J Cancer Surviv.

[33] Stegmann ME, Homburg TM, Meijer JM (2019). Correspondence between primary and secondary care about patients with cancer: a Delphi consensus study. Support Care Cancer.

[34] Heins M, Schellevis F, Rijken M (2012). Determinants of increased primary health care use in cancer survivors. J Clin Oncol.

[35] Krebber AM, Buffart LM, Kleijn G (2014). Prevalence of depression in cancer patients: a meta-analysis of diagnostic interviews and self-report instruments. Psychooncology.

[36] van Dipten C, Olde Hartman TC, Biermans MC, Assendelft WJ (2016). Substitution scenario in follow-up of chronic cancer patients in primary care: prevalence, disease duration and estimated extra consultation time. Fam Pract.

[37] Jeffery M, Hickey BE, Hider PN (2019). Follow-up strategies for patients treated for non-metastatic colorectal cancer. Cochrane Database Syst Rev.

[38] Wille-Jorgensen P, Syk I, Smedh K (2018). Effect of more vs less frequent follow-up testing on overall and colorectal cancer-specific mortality in patients with stage II or III colorectal cancer: the COLOFOL randomized clinical trial. JAMA.

[39] Liemburg GB, brandenbarg D, Berger MY (2021). Diagnostic accuracy of follow-up tests for detecting colorectal cancer recurrences in primary care: a systematic review and meta-analysis. Eur J Cancer Care (Engl).

